# Awareness, Treatment, and Control of Hypertension among the Adult Population in Burkina Faso: Evidence from a Nationwide Population-Based Survey

**DOI:** 10.1155/2021/5547661

**Published:** 2021-09-29

**Authors:** Kadari Cissé, Seni Kouanda, Yves Coppieters't Wallant, Fati Kirakoya-Samadoulougou

**Affiliations:** ^1^Centre de Recherche en Epidémiologie, Biostatistiques et Recherche Clinique, Ecole de Santé Publique, Université Libre de Bruxelles, Brussels, Belgium; ^2^Departement Biomédical et Santé Publique, Institut de Recherche en Sciences de la Santé, Ouagadougou, Burkina Faso; ^3^Institut Africain de Santé Publique, Ouagadougou, Burkina Faso

## Abstract

**Background:**

Hypertension is the leading cause of cardiovascular disease, particularly in low- and middle-income countries. Improved awareness of hypertension status can significantly increase early treatment, thereby reducing cardiovascular complications and premature death. This study aimed to report the prevalence of the awareness, treatment, and control of hypertension among the adult population in Burkina Faso.

**Method:**

We performed a secondary analysis of the first national population-based survey on common risk factors of noncommunicable diseases in Burkina Faso. It was a national representative cross-sectional survey among adults aged 25–64 years. Awareness of hypertension was defined by blood pressure ≥140/90 mmHg or a prior diagnosis by a health worker or the use of any antihypertensive drugs. A modified Poisson regression model using a generalized estimating equation was used to identify factors associated with awareness of hypertension.

**Result:**

A total of 4628 people with valid blood pressure measurements were considered. Of them, 828 had hypertension. Among people with hypertension, the prevalence of awareness was 17.5% (95% CI: 14.4%–21.1%), and 47.3% (95% CI: 37.6%–57.3%) of them had taken antihypertensive medications for their hypertension. One-third (35.5% (95% CI: 23.3%–49.9%)) of those who took medications had controlled hypertension. The prevalence of awareness was significantly higher among women (21.1% (95% CI: 16.4%–26.7%)) compared with men (13.8% (95% CI: 10.4%–17.9%)) (*p* = 0.019). The prevalence of awareness increased with increasing age and education level. Nearly one-third (29.3% (95% CI: 25.3%–33.6%)) of people with hypertension needed antihypertensive drug treatment.

**Conclusion:**

There was a poor level of awareness, treatment, and control of hypertension among adults in Burkina Faso. Effective control strategies to increase the screening of hypertension in primary care and at the community level are necessary in Burkina Faso.

## 1. Introduction

Hypertension is the leading cause of cardiovascular diseases (CVDs) worldwide, with at least 22.3% of CVDs being attributable to hypertension [1]. Nearly 9.4 million global deaths are attributable to hypertension and its complications [2]. In addition to heart complications such as ischemic heart disease, stroke, and heart failure, hypertension causes peripheral vascular diseases and many other vital organ impairments, such as renal or visual complications [3]. Most hypertension-related complications are preventable with lifestyle change strategies combined with antihypertensive drug therapy, which is known to substantially reduce the occurrence of future cardiovascular events [4]. However, hypertension remains an important global public health challenge. The number of cases was estimated to be 1.36 billion in 2010 and will reach 1.56 billion in 2025 [5, 6]. Low- and middle-income countries (LMICs) are mostly affected [6]. In sub-Saharan Africa (SSA), the number of cases of hypertension was estimated to be 130.2 million in 2010, only one-third of whom were aware of their hypertension; the number is projected to reach 216.8 million by 2030 [7]. Therefore, detection, treatment, and control are recognized as being of high priority for LMICs [6].

Hypertension is a silent and asymptomatic “killer,” since there are no symptoms in the early stage of this condition. Severe complications (e.g., stroke, heart attack, and kidney complications) might occur before symptoms appear. Despite the widespread availability of antihypertensive medications, the burden of hypertension and its complications is rising [6]. Unawareness of hypertension delays the early implementation of lifestyle change interventions and antihypertensive treatments to control the rising blood pressure. Improving the awareness among communities is crucial to reduce the delay of treatment initiation and to control the high blood pressure and prevent hypertension-related cardiovascular complications and mortality. In SSA, the public health response in this field is still low, with research findings showing that a high proportion of hypertensive individuals are unaware of their condition [7]. As there are often no symptoms in the early stage, the early detection of hypertension could be done through regular measurements of blood pressure in the community or during each visit to a health center. Few countries have yet to implement a program for hypertension awareness and control or provide guidelines to improve hypertension management at all levels of the health system [4]. Meanwhile, rapid urbanization, the “Western lifestyle,” and the increasing number of elderly adults have resulted in the urgency to describe the risk factors that delay the awareness, detection, and treatment of hypertension to help policymakers take effective prevention measures.

In Burkina Faso, there is a high prevalence of hypertension, estimated to be 18.5% [8]. This prevalence is lower than the mean of SSA (30.8%) [7]. National representative studies on awareness of hypertension are rare in SSA. Some national representative studies on awareness of hypertension were reported in Kenya (15.6%), Botswana (46%) [9], Guinea (24.8%) [10], and Nigeria (60%) [11]. As in many others countries in SSA, little is known about the awareness of hypertension among the adult population in Burkina Faso, while, in many countries across SSA, nationwide population-based strategies are urgently needed to improve the awareness of hypertension since it is projected that hypertension will be an important health priority. Many local studies have been done. Indeed, a local study in six health and demographic surveillance systems (HDSS) across SSA, including that of Nanoro (Burkina Faso), reported a prevalence of awareness of 39.9% in Nanoro, which seems to be lower than that reported in others regions (e.g., Kenya and South Africa) [12]. Another local study in the Kaya HDSS showed that the prevalence of awareness of hypertension was 26.8% [13]. These local studies are specific to their target population and might not be generalizable to Burkina Faso, since they were conducted among a small surveilled and controlled adult population. Accurate nationwide evidence on hypertension is needed to guide appropriate country-level policy response. The STEPwise survey, conducted in 2013 in Burkina Faso, offered an opportunity to assess the nationwide prevalence and factors that might influence the awareness of hypertension among the adult population in the country. Therefore, our study aimed to examine the prevalence of awareness of hypertension and its associated factors among adults in Burkina Faso. The results might help policymakers to improve hypertension control strategies to achieve national and global goals.

## 2. Materials and Methods

### 2.1. Study Type and Setting

This research was a secondary analysis of the last national survey on CVD risk factors conducted in 2013 in Burkina Faso. Burkina Faso is located in the SSA region in West Africa, covering a surface area of 272,960 km^2^ with 20,870,060 habitants in 2019 [14]. The country has two main urban cities (largest cities), namely, Ouagadougou (capital) and Bobo-Dioulasso (economic capital). Life expectancy at birth was 61.6 years in 2019 (https://data.worldbank.org/indicator/SP.DYN.LE00.IN?locations=BF). The proportion of the population living in urban areas increased (according to the results of the last national population census) from 12.7% in 1985 to 22.7% in 2006 [15]. The country comprises multiethnic groups (at least 60 ethnic groups) with different cultural practices in terms of demography, education, and alimentation [16, 17]. The ethnic groups are organized depending on the region, which creates natural clusters of sociocultural practices and behaviors related to CVD risk. The epidemiological profile is dominated by infectious diseases, with an increasing burden of noncommunicable diseases (NCDs), including CVDs, resulting in the country facing a double burden of disease and a progressive change of the pattern of diseases. Healthcare is provided by 2819 health facilities (2286 public and 533 private health facilities) grouped in 70 health districts. All health programs, including the NCDs program, are implemented by the health district lead teams. The health district is the first level of health program management.

### 2.2. Data Source: The STEP Survey

This unique national survey focused on common NCDs risk factors was carried out in 2013. It was a national and regional representative survey that used the World Health Organization (WHO) Stepwise approach and was a descriptive cross-sectional study including people aged 25–64 years, in which random sampling was performed. Cluster-stratified three-stage sampling was applied. In the first stage, 240 primary sampling unit enumeration areas (EAs) (or clusters) were randomly selected, proportionally to the number of households within each stratum (region and residence). The EA list provided from the fourth population and habitat census realized in Burkina Faso in 2006 (updated in 2010 during the Health and Demographic Survey) was used as the EA sampling base. A fixed number (i.e., 20) of the households were selected in each cluster after enumeration of the resident households. The Kish selection method was used to select a member of household to participate in the study. A total of 4800 persons were selected using the Kish approach, and from those, 4695 were interviewed; others declined to participate.

The stepwise survey collected much information about NCD risk factors and oral health. All risk factors were collected using the standardized WHO stepwise approach. Data on sociodemographic variables and behavioral cardiovascular risk factors were collected through direct interviews with household participants. The metabolic risk factors were collected through physical and biological measurements. Blood pressure was collected and measured by an electronic device (OMRON HEM-705 brand PC, Tokyo, Japan). The blood pressure was measured three times for each individual using the same device and by the same practitioner at the right arm after 15 min intervals rest. The participants with a valid measurements of blood pressure were considered in our analysis. The measurement of blood pressure was well described by Soubeiga et al. [8]. The mean of the last two readings was considered for the definition of hypertension.

### 2.3. Outcome Variable

Our analysis focused on hypertension, defined by blood pressure above or equal to 140 mmHg for systolic pressure and/or 90 mmHg for diastolic pressure or antihypertensive medication intake. The awareness of hypertension was measured during the survey by asking each participant the question, “Have you ever been told by a doctor, nurse, or other healthcare worker that you have hypertension (high blood pressure)?” [12]. The awareness of hypertension was defined by having hypertension and not being previously informed by a doctor, nurse, or other health worker. We also estimated the proportion of adults who had taken antihypertensive medications to treat their hypertension (the antihypertensive treatment was self-reported as formulated in the WHO STEPwise questionnaire). Among adults who took antihypertensive medication, we defined controlled hypertension as when the blood pressure was below 140/90 mmHg. We reported the proportion of hypertensive adults who had seen a traditional healer or taken traditional medicine. Additionally, we estimated the proportion of people who were not aware of their hypertension, even if they needed drug therapy, based on the WHO risk stratification approach for hypertension management [18].

### 2.4. Explanatory Variables

#### 2.4.1. Sociodemographic Variables

Based on previous studies using the same data [8, 19, 20] and other studies in SSA [21–24], we used the following variables as individual-level explanatory variables: age, sex, education, occupation status, and residence. Participants age was categorized into four groups (25–34, 35–44, 45–54, and 55–64 years). The sex variable was coded as 0 for “women” and 1 for “men.” For education level, attending formal school was considered and categorized into three groups (unschooled, primary, secondary and more). The occupation status was categorized into three groups (1 = “wage earner,” 2 = “self-employed,” and 3 = “jobless”). The residence of the participants was classified in two groups (1 = “urban” and 2 = “rural”).

#### 2.4.2. Other Cardiovascular Risk Factors

Tobacco use was focused on current smoking. Alcohol consumption in this study corresponded to usual alcohol consumption within 30 days given by the question, “Have you drunk an alcoholic drink in the last 30 days?” Optimal fruit and legume consumption corresponded to at least five portions of fruit and/or legumes consumed a day. For physical activity, we computed this category using the WHO's analysis guidelines: the variable was categorized into three groups (1 = “intensive physical activity,” 2 = “moderate physical activity,” and 3 = “low physical activity”). Body mass index (BMI) was classified into four groups, as recommended by WHO (1 = “underweight,” 2 = “normal weight,” 3 = “overweight,” and 4 = “obese”). The raised blood glucose was defined by capillary glycaemia as above or equal to 6.1 mmol/L. Based on the current guidelines, diabetes may be diagnosed based on plasma glucose criteria, either the fasting plasma glucose (FPG) value or the 2-h plasma glucose (2-h PG) value during a 75-g oral glucose tolerance test (OGTT), or A1C criteria [25]. Impaired fasting glucose (IFG) is defined as FPG levels from 100 to 125 mg/dL (from 5.6 to 6.9 mmol/L) [25]. So, it may be described based on capillary fasting blood glucose as “the raised fasting blood glucose” defined by capillary glycaemia above or equal to 6.1 mmol/L [26, 27]. Hypercholesterolemia was defined by capillary total cholesterol above or equal to 5.2 mmol/L.

### 2.5. Statistical Method

We first described the characteristics of the study population. We then evaluated the overall prevalence of awareness, included by sociodemographic and cardiovascular risk factors. For the treatment and control of hypertension, we reported the national prevalence without disaggregation by subgroups, since the numbers of treated and controlled hypertension cases were small. The chi-square test was used to check for differences in the prevalence of awareness by characteristics. A modified Poisson regression model using a generalized estimating equation (GEE) was implemented to derive the prevalence ratios (PRs) with robust variance while taking into account the clustering of observations. The univariate and multivariable GEE was applied to individual-level covariates using enumeration area identity as the clustering variable. PRs with 95% confidence intervals were calculated, and statistical significance at the 5% level (*p* < 0.05) was considered.

## 3. Results

### 3.1. Background Characteristics of the Study Population

**Figure 1:**
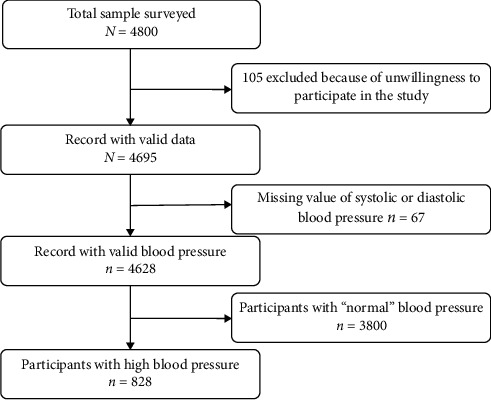
Flowchart of the study participants.

As shown in Figure 1, a total of 4628 participants had valid measurements of blood pressure, and 828 of them had hypertension as defined by WHO.

As shown in Table 1, half of the participants with hypertension were female, 22.2% were at least 55 years old, 10.5% had attended at least secondary school, and 61.9% were rural residents. Regarding cardiovascular behavioral, and biological profiles, 9.3% of the adults with hypertension were current smokers, nearly one-third (30.1%) were current alcohol drinkers, 11.5% were obese, and 8.7% had raised blood glucose (Table 1).

### 3.2. Prevalence of Awareness of Hypertension

Awareness was evaluated among the participants with hypertension.  Table 2 shows the prevalence of awareness of hypertension by sociodemographic, behavioral, and biological characteristics. The prevalence of awareness of hypertension was 17.5% (95% CI: 14.3–21.1). This prevalence was 21.1% (95% CI: 16.4–26.7) among women and 13.8% (95% CI: 10.4–17.9) among men. Regarding age groups, the prevalence of awareness of hypertension was 10.1% (95% CI: 5.9–16.8) for the 25–34 year olds, 9.6% (95% CI: 6.2–14.6) for the 35–44 year olds, 20.1% (95% CI: 14.5–27.2) for the 45–54 year olds, and 32.0% (95% CI: 24.9–39.9) for the 55–64 year olds. The prevalence of awareness of hypertension increased with education level—3.6% (95% CI: 10.7–17.1) for adults who had not attended formal school, 23.0% (95% CI: 15.2–33.2) for adults who had attended primary school, and 35.1% (95% CI: 25.6–46.0) for adults who had attended at least secondary school. One-quarter (24.3% (95% CI: 18.6–31.1)) of the adults who lived in urban areas were aware of their hypertension. This prevalence decreased to 13.2% (95% CI: 10.3–16.8) in rural areas. Regarding behavioral and biological profiles, the prevalence of awareness was 9.6% (95% CI: 4.2–20.6) among current smokers, 45.6% (95% CI: 31.1–61.0) among obese adults, and 26.3% (95% CI: 15.7–40.7) among adults with raised blood glucose (Table 2).

### 3.3. Factors Associated with Awareness of Hypertension

Table 2 presents the factors associated with awareness among hypertensive adults. After adjusting for all variables in Table 1, we found that age, education level, and obesity were significantly associated with awareness. Indeed, awareness increased with age: individuals aged 55–64 years had 3.5 times higher prevalence of the awareness compared to those who were aged 25–34 years. Individuals who had attended secondary school or more had 2.5 times (adjusted Prevalence Ratio (aPR): 2.52 (95% CI: 1.39–4.57)) higher prevalence of the awareness than those who had not attended formal school. Obese individuals had at least four times (aPR: 4.25 (95% CI: 1.81–10.02)) higher prevalence of the awareness than those who were underweight.

### 3.4. Treatment and Control of Hypertension

Among adults who were aware of their hypertension, 47.3% (95% CI: 37.6–57.3) reported antihypertensive medication prescribed by a doctor or other health worker. One-third (35.5% (95% CI: 23.3–49.9)) of those who took medication had controlled their hypertension. Nearly one-third (29.3% (95% CI: 25.3–33.6)) of people who were not aware of their hypertension needed antihypertensive drug treatment according to WHO guidelines for cardiovascular risk management. This proportion was 27.3% (95% CI: 21.9–33.3) for women and 31.1 (95% CI: 25.4–37.4) for men (*p*=0.35). In urban areas, this figure was 34.1% (95% CI: 27.3–41.5), and for rural areas, it was 26.7% (95% CI: 21.9–31.2) (*p*=0.097). We noted that 13.9% (95% CI: 8.2–22.6) of the adults who were aware their hypertension had seen a traditional healer, and 11.6% (95% CI: 6.7–19.6) were taking herbal or traditional remedy.

## 4. Discussion

### 4.1. Key Findings

This study provides the country-level representative prevalence of awareness of hypertension among the adult population in Burkina Faso, which is one of the lowest-income countries in the world with a high prevalence of hypertension and of other cardiovascular risk factors, as shown by the first STEPwise survey and its related published papers [8, 19, 20, 28]. In this study, we found that only 17.5% of hypertensive adults were aware of their condition. The prevalence of awareness of hypertension was higher among the elderly population, people with more education, and those who were obese. Nearly half (47.3%) of the hypertensive adults with awareness of their condition had received treatment for their hypertension, and only one-third (35.5%) of them had their hypertension controlled.

### 4.2. Prevalence of Awareness

The prevalence of awareness in our population was lower compared to the overall prevalence in SSA reported by Adeloye and Basquill [7] in a systematic review, which was estimated to be 33.7%. The overall prevalence of awareness of hypertension in low-income countries (LICs) was estimated to be 40.8% [29]. A recent study in Kenya using the 2015 Kenya STEP survey data reported a prevalence of awareness of hypertension of 15.6% in Kenya [30]. Using the STEP survey, the prevalence of awareness was 24.8% in 2016 in Guinea [10]. In Nigeria, a nationwide survey in 2017 showed that six out of every 10 hypertensive patients (60%) were aware of their status [11]. In Botswana, in 2014, 46% of hypertensive adults were aware of their hypertensive status [9]. These results in SSA show an important variability in the awareness of hypertension across countries. However, compared to high- and middle-income countries, the overall prevalence of awareness of hypertension remains lower in LICs [29]. Hypertension detection is also lower in LICs, despite its prioritization by many global organizations. The control of hypertension is also recognized to be lower in such countries, despite the availability of effective antihypertensive medications [29]. This situation might be explained by the fact that few individuals in such countries have access to routine blood pressure checks due to poor access to healthcare and hypertension screening programs [29]. The lower rate of awareness of hypertension reported in this study reflects the poor capacity of the health system of the country to manage hypertension. Hypertension remains underdiagnosed among adults in Burkina Faso, despite the availability of self-measurement tools, and it is still poorly screened, even in healthcare centers [30]. Studies in HDSS have reported a higher prevalence of awareness of hypertension (39.9% in Nanoro [12] and 26.8% in Kaya [13]) compared to ours, because the populations of HDSS are subjects of many and regular medical research efforts, which might contribute to increasing their awareness of their health conditions. Regarding the negative effects of unawareness of hypertension on the current rising burden of cardiovascular diseases, it is crucial to pay attention to hypertension screening among the adult population in Burkina Faso. Task sharing with nonphysician healthcare workers (including community health workers) for the screening of hypertension might be an effective solution, since a recent meta-analysis reported the benefit of this intervention on the effective management of blood pressure [31, 32].

### 4.3. Associated Factors of Awareness

This study reported that, after adjusting for sociodemographic variables and cardiovascular risk factors, the awareness of hypertension was higher among the elderly population, obese adults, and people with more education, which is in accordance with the research findings in the context of low-income countries. Many studies have reported a high prevalence of awareness of hypertension among elderly and obese people [3, 10, 11, 33, 34]. This finding might be explained by the higher utilization of healthcare services and frequent contact with health centers due to the frequent deterioration of their health [33]. The frequent contact with health centers might also explain the high prevalence of awareness of hypertension among women, even if this relationship disappeared when adjusting for other sociodemographic and risk factor variables. Low-level education was associated with a lower rate of awareness of hypertension, and this finding is consistent with the results of the Prospective Urban Rural Epidemiology (PURE) study in low-income countries that highlighted a lower rate of awareness of hypertension among people with primary or no education [29]. The lack of knowledge about hypertension and its complications [29], low socioeconomic status, and access to healthcare might explain the high rate of unawareness among people with no education, suggesting a future high burden of hypertension-related cardiovascular disease among this population. In our study, the awareness of hypertension was higher in urban areas. Evidence from the PURE study shows that, in low-income countries, the awareness of hypertension is higher in urban areas compared to rural areas [29]. Meanwhile, these differences were not highlighted in middle- and high-income countries, suggesting inequities in access to hypertension management healthcare in LICs such as Burkina Faso.

### 4.4. Treatment and Control of Hypertension

This study reported that half of the participants aware of their hypertension did not take antihypertensive medication, and nearly two-thirds of them had not controlled their hypertension. This finding is consistent with studies conducted in many LIC contexts [10, 29, 33]. The lower rate of treatment of hypertension in LICs, including those reported in this study, is due—as noted by Yaya Bocoum and Hartwig to the low access to hypertension-related healthcare (distance to health facilities, cost to see physicians, cost of medication, etc.), since universal healthcare coverage or health insurance does not usually include severe and chronic illnesses such as hypertension [35]. This situation may encourage herbal or traditional medicine use to treat hypertension and delay an effective management of hypertension. In SSA, a review showed that 47.5% of adults with hypertension had concomitantly used herbal or traditional medicine and allopathic medicine [36]. A similar proportion (40,3%) was reported by Camara et al. [10] in Guinea in 2016 (using STEPwise survey data). Our study noted that one-tenth hypertensive adults had taken traditional medicine. Our study also noted that nearly one-third of hypertensive adults needed drug treatment, according to the WHO guidelines for cardiovascular risk management [18]. The 10-year cardiovascular risk strata were defined using the Framingham global risk score, since this risk score is the only risk score that covers a large number of complications pertaining to hypertension (including ischemic heart disease, stroke, peripheral vascular disease, heart attack, or heart failure) [37]. This risk score is also commonly used in clinical practice in Burkina Faso [38, 39]. A new approach to address the gap in hypertension treatment is needed. The approach could include the development of national guidelines, the strengthening of antihypertensive medication availability and affordability, the training of healthcare providers, and the shifting of the screening and follow-up of treatment of hypertension to nonphysician health workers or community health workers, since these categories of health workers have shown their effectiveness in hypertension management [31, 32].

### 4.5. Strengths and Limitations

The main limitation of this study was the measurement of blood pressure in a single visit, which might have overestimated the number of individuals with high blood pressure. The ambulatory monitoring of blood pressure is recognized to predict the risk for morbidity more accurately than a single measurement of blood pressure [40]. The findings presented in this study are specific to adults aged 25–64 years. They may not be extrapolated to adults under 25 years or over 64 years old. In addition, due to its cross-sectional design, the findings of this study might not be considered to derive the causality. Even with the lower level of controlled hypertension in this study, this finding might be affected by the patients' previous experience of medications for other conditions, which might have contributed to their adherence to antihypertensive treatment. The main strength of this study is that it was a nationally representative estimation of awareness of hypertension. We recognize that seven years have elapsed since the first STEPwise survey in Burkina Faso, and the data used for this analysis need to be updated. However, our study provides baseline data for future assessment of the impact of the national noncommunicable disease program, which was drafted in 2016 [41].

## 5. Conclusion

This study is the first nationally representative estimation of awareness of hypertension and its treatment and control in Burkina Faso. The findings suggest a lower prevalence of awareness, treatment, and control, as shown in many other previous studies in low-income countries. We also noted that the elderly, those with more education, and obese people had a higher prevalence of awareness of hypertension. This study shows a national picture of hypertension, before starting any efforts to manage NCDs in the country, which might offer an opportunity to better understand the impact of the national NCD program initiated in 2016 on the hypertension burden. While waiting for a new STEPwise survey in Burkina Faso, these findings might guide policymakers for effective management of hypertension in the country, which might start by better screening people with hypertension through real task sharing with nonphysicians or community health workers. Nevertheless, more studies should be needed to show the best way to improve awareness, treatment, and control of hypertension in the low resource setting, since hypertension is rising in such context and suggesting a future epidemic of hypertension-related complications like cardiovascular diseases.

## Figures and Tables

**Table 1:** Sociodemographic, behavioral, and biological risk factor profiles of the study population.

Characteristics	Number	Percentage
All participants with hypertension	828	100.0
*Sex*		
Women	389	50.4
Men	439	49.6

*Age group, years*		
25–34	230	25.1
35–44	211	26.5
45–54	201	26.2
55–64	186	22.2

*Marital status*		
Single	160	17.6
Married	668	82.4

*Completed level of education*		
No formal school	612	72.5
Primary school	128	17.0
Secondary or higher	88	10.5

*Profession*		
Wage earner	66	8.1
Self-employed	564	64.8
Jobless	198	27.1

*Residence*		
Urban	256	38.1
Rural	572	61.9

*Current smoker*		
No	743	90.7
Yes	85	9.3

*Current drinker*		
No	569	69.0
Yes	259	31.0

*Fruit and vegetable intake*		
<5	788	95.6
≥5	40	4.4

*Physical activity*		
Intense	451	54.2
Moderate	210	25.0
Low	167	20.8

*BMI class*		
Underweight	74	8.7
Normal	502	58.5
Overweight	163	21.3
Obese	89	11.5

*Raised blood glucose*		
No	753	91.3
Yes	75	8.7

*Hypercholesterolemia*		
No	777	93.2
Yes	51	6.8

BMI: body mass index.

**Table 2:** Prevalence and results of multivariable modified Poisson regression of hypertension awareness among hypertensive adults in Burkina Faso.

Characteristics	Awareness of hypertension						
	*N*	*n*	Prevalence % [95% CI]	Crude PR [95% CI]	*p* value	Adjusted PR [95% CI]	*p* value
All	828	143	17.5 [14.3–21.1]				
*Sex*					0.025		0.35
Women	389	82	21.1 [16.4–26.7]	1		1	
Men	439	61	13.8 [10.4–17.9]	0.68 [0.48–0.95]		0.82 [0.55–1.23]	

*Age group, years*					<0.001		<0.001
25–34	230	20	10.1 [5.9–16.8]	1		1	
35–44	211	23	9.6 [6.2–14.6]	1.18 [0.66–2.11]		1.17 [0.63–2.13]	
45–54	201	45	20.1 [14.5–27.2]	2.18 [1.29–3.68]		2.34 [1.37–4.01]	
55–64	186	55	32.0 [24.9–39.9]	3.01 [1.81–5.00]		3.59 [2.10–6.14]	

*Marital status*					0.68		0.87
Single	160	32	19.6 [12.6–29.2]	1		1	
Married	668	111	17.0 [13.7–20.9]	0.92 [0.61–1.39]		1.03 [0.68–1.57]	

*Completed level of education*					<0.001		0.009
No formal school	612	85	13.6 [10.7–17.1]	1		1	
Primary school	128	25	23.0 [15.2–33.2]	1.34 [0.84–2.11]		1.37 [0.84–2.25]	
Secondary or higher	88	33	35.1 [25.6–46.0]	2.45 [1.57–3.79]		2.52 [1.39–4.57]	

*Profession*					0.006		0.58
Wage earner	66	20	22.2 [14.2–32.8]	1		1	
Self-employed	564	76	13.4 [10.3–17.3]	0.51 [0.29–0.86]		0.82 [0.42–1.63]	
Jobless	198	47	25.7 [19.3–33.4]	0.84 [0.49–1.47]		1.04 [0.54–1.99]	

*Residence*					0.029		0.22
Urban	256	62	24.3 [18.6–31.1]	1		1	
Rural	572	81	13.2 [10.3–16.8]	0.62 [0.40–0.95]		1.35 [0.83–2.21]	

*Current smoker*					0.043		0.68
No	743	136	18.3 [15.0–22.1]	1		1	
Yes	85	7	9.6 [4.2–20.6]	0.46 [0.21–0.97]		0.85 [0.37–1.89]	

*Current drinker*					0.75		0.39
No	569	101	18.0 [14.2–22.6]	1		1	
Yes	259	42	16.2 [11.8–21.8]	0.94 [0.65–1.36]		0.84 [0.57–1.25]	

*Fruit and vegetable intake*					0.21		0.14
<5	788	132	17.1 [13.9–20.8]	1		1	
≥5	40	11	25.2 [12.5–44.3]	1.51 [0.78–2.90]		1.62 [0.85–3.08]	

*Physical activity*					0.068		0.72
Intense	451	63	13.7 [10.4–17.9]	1		1	
Moderate	210	41	20.6 [14.6–28.4]	1.63 [0.91–2.09]		1.14 [0.75–1.72]	
Low	167	39	23.5 [16.9–31.7]	1.63 [1.06–2.50]		0.95 [0.60–1.51]	

*BMI class*					<0.001		<0.001
Underweight	74	7	9.5 [4.4–19.5]	1		1	
Normal	502	61	11.5 [8.7–15.0]	1.25 [0.58–2.69]		1.57 [0.71–3.46]	
Overweight	163	38	21.9 [16.1–29.1]	2.24 [1.01–4.97]		2.67 [1.17–6.11]	
Obese	89	37	45.6 [31.1–61.0]	4.09 [1.84–9.12]		4.25 [1.81–10.02]	

*Raised blood glucose*					0.45		0.80
No	753	125	16.6 [13.3–20.5]	1		1	
Yes	75	18	26.3 [15.7–40.7]	1.23 [0.72–2.11]		0.93 [0.54–1.61]	

*Hypercholesterolemia*					<0.001		0.26
No	777	122	42.7 [27.9–58.9]	1		1	
Yes	51	21	15.6 [12.9–18.9]	0.42 [0.26–0.68]		0.74 [0.43–1.26]	

*N*: total number of participants with hypertension; *n*: number of participants who were aware of their hypertension; PR: prevalence ratio; CI: confidence interval.

## Data Availability

The data used to support the findings of this study are available at the Ministry of Health upon request to Bicaba Brice (bicaba_brico@yahoo.fr) or Zoma Torez (torezo2000@yahoo.fr). All survey materials are available on the WHO website (https://extranet.who.int/ncdsmicrodata/index.php/catalog).
